# Mechanical characteristics and stability analysis of surrounding rock reinforcement in rectangular roadway

**DOI:** 10.1038/s41598-022-26773-z

**Published:** 2022-12-23

**Authors:** Shuan-Cheng Gu, Pan Wang, Chao-Fan Yang

**Affiliations:** 1 School of Architecture and Civil Engineering, Xi’an University of Science and Technology, Xi’an, 710054 China; 2 School of Urban Construction, Xi’an Kedagaoxin University, Xi’an, 710109 China

**Keywords:** Civil engineering, Carbon capture and storage, Mathematics and computing

## Abstract

The stability of surrounding rock with bolt support depends on the stability within the reinforcement range. To understand the reinforcing mechanism of a rectangular roadway bolt fully and accurately, a quantitative method for evaluating the stability of the surrounding rock of a rectangular roadway must be developed. First, a roof beam model of a rectangular tunnel is established according to the deformation law of surrounding rock. Based on the elastic–plastic theory, the deflection calculation formula can be derived, and the ultimate load of the roof beam can be obtained under the plastic state without support. Second, based on the reinforcement effect of bolts, a model of a surrounding rock reinforcement body is established, the physical and mechanical properties of this body are deduced, and a method for evaluating the stability of surrounding rock is derived. Finally, by considering actual engineering cases, the theoretical calculation results of surrounding rock deformation are compared with the numerical simulation and field monitoring results. Moreover, the influence of different parameters of the bolt support on the mechanical characteristics and stability of reinforcement is investigated. The results show that the theoretical calculations approximate the numerical simulation and field monitoring values, thus verifying the rationality of the theory. The physical and mechanical properties and stability of the surrounding rock reinforcement body are considerably affected by changes in bolt length and spacing. The anchor design must apply the following principle: the bolt must either be long and sparsely spaced or short and densely spaced. The theory presented in this paper provides a relatively simple and fast quantitative calculation method for the study of the surrounding rock stability of bolt-supported rectangular roadways.

## Introduction

When the stress state exceeds the elastic peak state of surrounding rock, the roof undergoes plastic deformation and is considerably displaced. Rock bolt support is widely used as roadway support because of its fast construction speed, satisfactory support effect, and low economic cost^1–3^. Many engineering applications show that the use of bolts is an effective approach to support and reinforce roadways, limit surrounding rock deformation, and improve surrounding rock strength^4–6^.The analysis of the physical and mechanical properties of surrounding rock with bolt reinforcement and bolt support mechanism has been the focus of research ^7–11^. Kim SH et al. ^12^reported that bolt support improved the cohesion of surrounding rock. They further presumed that the improvement effect on the internal friction angle of the surrounding rock is not evident. Bobet et al. ^13^ developed a mechanical model for coupling bolts and surrounding rock and then analyzed the stress and deformation expressions of the surrounding rock considering different bolt support parameters. Indraratna B^14^ and Osgoui RR^15^ used a bolt density factor to analyze the reinforcement effect of bolts on surrounding rocks and subsequently deduced a deformation expression for the surrounding rock with bolt support. Gu SC et al.^16^ considered a new reinforcement structure for surrounding rock and a composite of circular roadway bolt; then, they obtained the physical and mechanical parameter expressions for the reinforcement body of the surrounding rock. Meng Q et al.^9^ derived a new elastoplastic analytical solution for a tunnel without support and applied a homogenization method to obtain the equivalent strength parameters when support was provided. Gao JM et al.^17^ evenly distributed the shear force exerted by surrounding rock on a micro-segment of a full-length bond bolt. Subsequently, they deduced the expression for surrounding rock stress and displacement under the support of a full-length bond bolt in a circular roadway.

The aforementioned scholars analyzed the solution of the surrounding rock deformation of a circular roadway and the action mechanism of surrounding rock reinforcement with anchor bolts through different research methods. Although numerous results have been collected, shortcomings remain, as follows. (1) Rectangular roadways are more widely used in practical engineering; however, the deformation law of surrounding rock of circular roadways differs from that of rectangular roadways. Therefore, the study of the bolt support and reinforcement of circular roadways is not suitable for rectangular roadways^18–21^. (2) The traditional support theory regards bolts as a distinct supporting structure, and their reinforcement effect is ignored. (3) When the reinforcement effect of a bolt is considered, only its physical parameters are presumed to be distributed evenly to those of surrounding rock, and the coupling between the bolt and surrounding rock is ignored.

Accordingly, a roof beam model of a tunnel is formulated based on the deformation law of surrounding rock of a rectangular tunnel roof and previous studies^22,23^. The compressive strength of coal is greater than the tensile strength; hence, the deflection calculation formula and ultimate load of a roof beam can be determined in the plastic state without support based on the elastic–plastic theory^24^. By considering the change in the mechanical state of surrounding rock caused by the bolt, a model of the reinforcement body of surrounding rock was established. Furthermore, the physical and mechanical parameters of the surrounding rock reinforcement body and the method for evaluating the stability of surrounding rock were derived. The reliability of the theory is verified by actual engineering site monitoring and the FLAC3D numerical simulation software.

## Analysis of mechanical characteristics of surrounding rock of roadway roof without support

### Development of roof beam model

**Figure 1:**
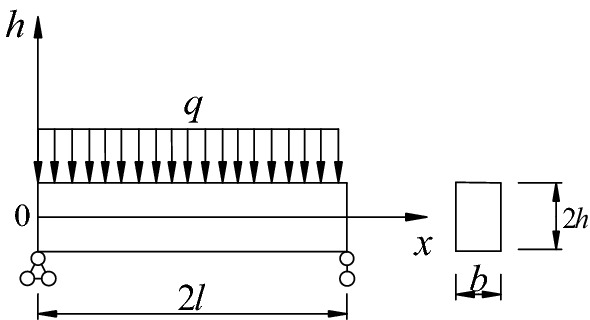
Roof beam model of rectangular roadway.

According to the deformation law of surrounding rock of rectangular roadway, the roadway roof may be considered as a simply supported beam model, and the beam cross-section can be approximately simplified as a rectangle. The length and height of the roof beam are calculated as $2l$ and $2h$, respectively; the width of the beam is $b$; and the beam bears the dead weight load, $q$, of the overlying rock mass. The established model is shown in Fig. 1.

Assumptions made in the structural model are as follows:

- (1) An analysis of the plane strain problem is conducted using the roadway section after excavation.
- (2) An ideal elastic–plastic material with different tensile and compressive strengths and continuous, homogeneous, isotropic properties.
- (3) The thickness of the roadway is a unit in the axial direction.
- (4) The surrounding rock is broken as a result of the concentrated stress in the corner of the road, and the corner therefore serves as a support for the hinge.
- (5) It is primarily appropriate for horizontal layered rocks.

### Analysis of elastic–plastic state of roof beam

To derive the deflection equation of the beam under the elastic–plastic state, first, the elastic–plastic evolution process of the beam section is analyzed, and the bending moment corresponding to the beam section at different stages is obtained. Then, the deflection equation of the beam in the elastoplastic state is obtained based on the assumption that a plane section remains plane.

**Figure 2:**
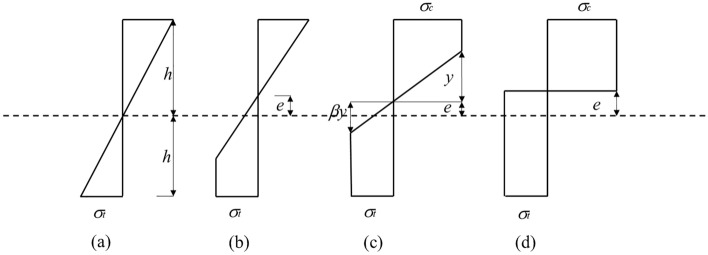
Elastic–plastic evolution diagram of roof beam section: (**a**) elastic stage; (**b**) first elastic–plastic stage; (**c**) second elastic–plastic stage; (**d**) plastic stage.

The stress distribution on the roof beam section depends on the plastic stage of coal, as shown in Fig. 2. When the maximum stress on the tensile side of the beam section is less than the ultimate tensile strength of the surrounding rock, the entire section is in an elastic state; the corresponding normal stress distribution on the section is shown in Fig. 2a. When the maximum stress on the tensile side of the beam section reaches the ultimate tensile strength of the surrounding rock, the strain on this section continues to increase. However, the stress stops increasing, and the plastic zone on the tensile side of the beam section gradually expands. At this time, the neutral axis begins to deviate to ensure that the sum of normal stresses on the beam section is zero, as shown in Fig. 2b. When the maximum stress on the compression side of the beam section reaches the ultimate compressive strength of the surrounding rock, this side also enters the plastic state, as shown in Fig. 2c. As the stress in the beam section further increases, the compression side eventually enters the plastic state; hence, the entire beam section is in the plastic state, as shown in Fig. 2d.

In the figure, $\sigma_{t}$ is the ultimate tensile strength of surrounding rock; $\sigma_{c}$ is the ultimate compressive strength of surrounding rock; $\beta$ is the ratio of the tensile yield strength to the compressive yield strength of surrounding rock; $e$ is the offset distance of the neutral axis; $y$ is the distance between the junction of the elastic–plastic zone at the upper layer of the beam and neutral axis; and $\beta y$ is the distance between the junction of the elastic–plastic zone at the lower layer of the beam and neutral axis.

When the beam section is in the elastic state, as shown in Fig. 2a, the maximum tensile stress of the section is equal to the ultimate tensile strength of the surrounding rock. The elastic ultimate bending moment of the beam section is as follows^25^:

1 $$ {\text{M}}_{{\text{e}}} = \frac{{{\text{2bh}}^{{2}} {\upsigma }_{{\text{t}}} }}{{3}}{,} $$

where $M_{e}$ is the ultimate bending moment of the beam section in the elastic state.

The midspan bending moment of a simply supported beam under a uniform overburden load is as follows^26^:

2 $$ M_{ke} = \frac{{ql^{2} }}{{2}}, $$

where $M_{ke}$ is the midspan bending moment of a simply supported beam under a uniformly distributed load.

When the beam section is in the second elastic–plastic stage, as shown in Fig. 2c, the following equation can be obtained according to the sum of stresses on the beam section being zero:

3 $$ \sigma_{c} (h - e - y) + \frac{{\sigma_{c} }}{2}x = \sigma_{t} (h + e - \beta y) + \frac{{\sigma_{t} }}{2}\beta y. $$

After simplifying Eq. (3), the centrifugal distance can be obtained as:

4 $$ e = \frac{(1 - \beta )}{2}\left( {\frac{2h}{{1 + \beta }} - y} \right). $$

The relationship between stress and bending moment is analyzed based on the stress distribution diagram shown in Fig. 2c. The calculated combined stress is applied to its shape center to determine the moment of this stress with respect to the neutral axis. After simplification, the following is obtained:

5 $$ M_{ep} = M_{e} \left[ {\frac{3}{4} + \frac{3}{4\beta } - \frac{{y^{2} }}{{{4}h^{2} }}\left( {\frac{1}{\beta } + \beta^{2} } \right)} \right], $$

where $M_{ep}$ is the bending moment of the beam section under the elastic–plastic state.

When $y = 0$, the entire beam section is in the plastic state, as shown in Fig. 2d. The bending moment of the beam section can be obtained using Eq. (5):

6 $$ M_{p} = \frac{{{3}M_{{\text{e}}} }}{{4}} \left( {1 + \frac{1}{\beta }} \right), $$

where $M_{p}$ is the bending moment of the section in the plastic state.

The bending moment distribution rule of simply supported beams under uniform load is as follows:

7 $$ M = M_{p}  \left( {1 - \frac{{(l{ - }x)^{2} }}{l}} \right). $$

According to Eq. (7) and using the assumption that a plane section remains plane, the deflection equation of the beam in the plastic state can be derived using Eqs. (8) and (9):

8 $$  {0} \le x \le {0}{\text{.08}}l \cup {1}{\text{.92}}l \le x \le {2}l,\,\omega  =  - \frac{{qlx^{3} }}{{4Ebh^{3} }} + \frac{{qx^{4} }}{{16Ebh^{3} }} + 0.0{3} \cdot \left( {\ln 0.31l - 1.09} \right)\frac{{ql^{{3}} x}}{{bEh^{3} }}, $$

9 $$  {0}{\text{.08}}l \le x \le {1}{\text{.92}}l,\omega = - {4}{\text{.64}}\frac{{ql^{3} x}}{{bEh^{3} }}(\ln x - 1 - \ln l), $$

where $\omega$ is the deflection of the beam midspan section in the plastic state ;$E$ is the elastic modulus of the surrounding rock .

The deflection of the middle section of the beam span in the plastic state can be obtained by substituting $x = l$ (i.e., the middle section of the beam span) into Eq. (9):

10 $$ \omega_{p} = {4}{\text{.64}}\frac{{ql^{4} }}{{bEh^{3} }}, $$

where $\omega_{p}$ is the deflection of the beam midspan section under the plastic state.

According to Eqs. (1), (2) and (6), the ultimate load that the beam can bear when the midspan section of the beam is in the plastic state is as follows:

11 $$ q * = \frac{{bh^{2} \sigma_{t} \,\left( {{1} + \frac{{1}}{\beta }} \right)}}{{l^{2} }}, $$

where $q *$ is the ultimate load that can be borne by the midspan section of the beam in the plastic state.

It is shown that the relationship between the ultimate compressive strength of the surrounding rock and the beam height under the plastic limit state is as follows when the maximum normal stress on the beam at mid-span does not exceed the ultimate compressive strength of the surrounding rock.

12 $$ \sigma_{\max }  = \frac{{M_{{\text{p}}} }}{{(h_{{\text{p}}} - e) \cdot b \cdot \left( {\frac{{h_{{\text{p}}} - e}}{2} + \frac{{h_{{\text{p}}} + e}}{2}} \right)}} = \frac{{187ql^{2} }}{{5b\left( {2h_{{\text{p}}} } \right)^{2} }} \le \sigma_{{\text{c}}} , $$

where $2h_{{\text{p}}}$ is beam height corresponding to the plastic limit state of the roof beam.

Therefore, the beam height for the roof beam in the plastic limit state is:

13 $$ 2h_{{\text{p}}} = \sqrt {\frac{{187ql^{2} }}{{5b\sigma_{{\text{c}}} }}} . $$

There is an appearance of a plastic hinge in the roof beam model when $2h < 2h_{{\text{p}}}$. Consequently, the unsupported roof beam model has a roof height $2h = 2h_{{\text{p}}}$. In the supported roof beam model, since the reinforcement body support the load collectively, the roof beam model height is taken as the anchor length.

## Analysis of mechanical properties of surrounding rock reinforcement body

### Determination of elastic modulus of surrounding rock reinforcement body

**Figure 3:**
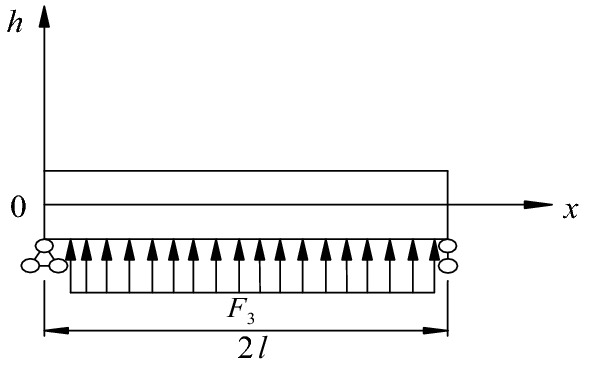
Schematic of equivalent uniform load on surrounding rock of roadway roof.

One of the reinforcement effects of bolt support is the increase in the elastic modulus of surrounding rock in the reinforcement area, thus increasing the strength of surrounding rock. The surrounding rock reinforcement zone with anchor rods is shown in Fig. 3. The supporting force of anchor rods on surrounding rock is equivalent to a uniform load acting on a semi-plane body. Hence, the deformation of the surrounding rock constrained by the anchor rods in the beam span is as follows^27^:

where $F{}_{{3}}^{{}}$ is the equivalent supporting force of the bolt on the surrounding rock.

14 $$ \begin{aligned} u & & = & \frac{{2F_{3} (1 - \mu^{2} )}}{\pi El}\int_{0}^{l} {\ln \frac{{{2}l}}{r}} dr \\ & &  = & \frac{{{3}{\text{.39}}F_{3} (1 - \mu^{2} )}}{\pi E}, \\ \end{aligned} $$

where $u$ is the deformation amount of surrounding rock restrained by anchor rod in middle of roadway;$\mu$ is Poisson’s ratio of surrounding rock.

15 $$ F_{{3}} = \frac{{N + \frac{\Delta w}{{l_{g} }}AE_{{\text{g}}} }}{S}, $$

where $\Delta w$, $l_{g}$, $A$, $N$, and $E_{g}$ are the elongation, length, cross-sectional area, pre-tightening force, and elastic modulus of the bolt, respectively; $S$ is the bolt spacing.

16 $$ \Delta w = {4}{\text{.64}}\frac{{ql^{4} }}{{bEh^{3} }}{ - }u. $$

The elastic modulus of the surrounding rock after bolt reinforcement can be derived according to the reduction of the longitudinal deformation of the surrounding rock:

17 $$ E_{R} = \frac{{{4}{\text{.64}}ql^{4} \pi E}}{{{4}{\text{.64}}q\pi l^{4} - {3}{\text{.39}}bh^{3} F_{3} (1 - \mu^{2} )}}, $$

where $E_{R}$ is the elastic modulus of the surrounding rock reinforcement body.

### Determination of cohesion of surrounding rock reinforcement body

**Figure 4:**
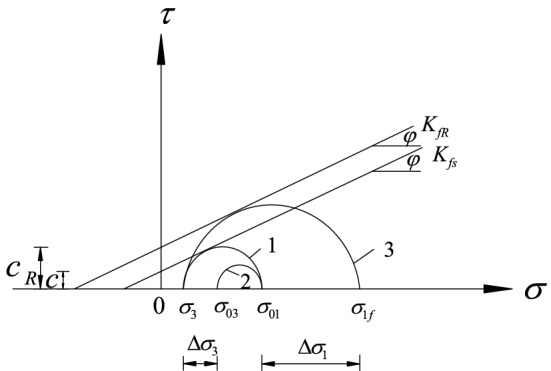
Mohr’s envelope of rock strength.

The initial stress state of the surrounding rock is assumed as ($\sigma_{{{01}}}$,$\sigma_{{{03}}}$), as shown in circle 2 (Fig. 4); the ultimate stress state has not yet been reached at this time. For the stress to reach ultimate stress state, it must be ($\sigma_{{{01}}}$,$\sigma_{{3}}$), as shown in circle 1 (Fig. 4). At this point, the surrounding rock undergoes ultimate deformation because the stress is reduced to the ultimate equilibrium state.

If the surrounding rock is in the($\sigma_{{{01}}}$,$\sigma_{{3}}$) limit equilibrium state, as shown in circle 1(Fig. 4). The surrounding rock reinforcement body is far from the limit equilibrium state when the bolt is added. Assuming the stress state recover from the Circle 1 to Circle 2 in Fig. 4, the effect of adding bolts is then equivalent to imposing a vertical stress increment $\Delta \sigma_{3}$ on surrounding rock^28^.

Where $K_{fs}$ is strength envelope tangent to circle 1; $\phi$ is the internal friction angle; $K_{fR}$ is the strength envelope tangent to circle 3; ($\sigma_{{{01}}}$,$\sigma_{{{03}}}$) is the initial stress state of the surrounding rock; ($\sigma_{{3}}$,$\sigma_{1f}$) is the limit equilibrium stress state of the surrounding rock; $C$ is the cohesion of the surrounding rock; and $C_{R}$ is the cohesion of the surrounding rock after reinforcement.

Because the anchor rod is added to the surrounding rock, vertical stress is increased such that the added solid remains in equilibrium in the ($\sigma_{{3}}$,$\sigma_{{{01}}}$) stress state. To produce a limit equilibrium state, $\sigma_{{{01}}}$ must be increased to $\sigma_{1f}$ (i.e., the stress state shown in circle 3 (Fig. 4)). According to Yang SS et al.^29^, the internal friction angle of the surrounding rock after reinforcement remains approximately equal to that when an anchor is not provided. As shown in Fig. 4, under the same vertical pressure, the addition of the solid increases the lateral stress, $\Delta \sigma_{{1}}$, which exceeds that of unreinforced rock. The effect of introducing an anchor rod is equivalent to adding a cohesive force, *C*_*R*_–*C*, to the surrounding rock or increasing its ability to withstand the lateral load, $\Delta \sigma_{{1}}$, than the unreinforced rock.

**Figure 5:**
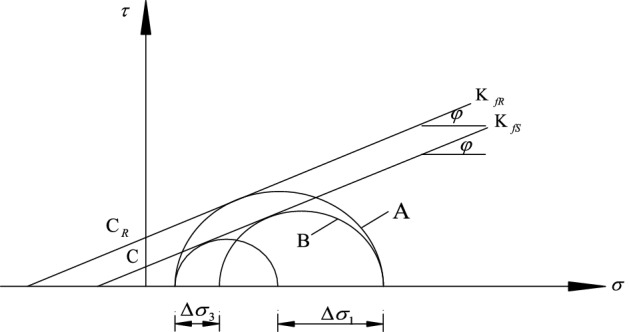
Mohr’s stress circle of surrounding rock reinforcement body.

In terms of improving the performance of the surrounding rock, the addition of an anchor rod corresponds to increasing the cohesive force of the surrounding rock. The surrounding rock reaches the limit equilibrium state when the stress is as shown in circle A (Fig. 5). According to the Mohr–Coulomb criterion of rock failure, the limit balance is as follows:

18 $$ \sigma_{1f} = \sigma_{3} \frac{1 + \sin \phi }{{1 - \sin \phi }} + 2\frac{{C_{R} \cos \phi }}{1 - \sin \phi }. $$

From the perspective of modifying the mechanical state of the surrounding rock, the addition of the anchor rod is equivalent to applying a vertical stress increment to the surrounding rock. At this time, the surrounding rock reaches the limit equilibrium state shown in circle B (Fig. 5). The limit equilibrium is obtained according to the Mohr–Coulomb criterion of rock failure:

19 $$ \sigma_{1f} = \left( {\sigma_{3} + \Delta \sigma_{3} } \right)\frac{1 + \sin \phi }{{1 - \sin \phi }} + 2\frac{C\cos \phi }{{1 - \sin \phi }}, $$

where $\Delta \sigma_{{3}}$ is the vertical stress increment imposed by the bolt on the surrounding rock and is equal to $F_{{3}}$.

According to Eqs. (18) and (19), the cohesion of the surrounding rock reinforcement body is as follows:

20 $$ C_{R} = \frac{{\Delta \sigma_{{3}} \left( {{1} + \sin \phi } \right)}}{2\cos \phi } + C. $$

### Determination of Poisson’s ratio of surrounding rock reinforcement body

Based the definition of the shear modulus of surrounding rock and the relationship between shear modulus, elastic modulus, and Poisson's ratio, the following can be derived^27^:

21 $$ G = \frac{\tau }{\gamma } = \frac{E}{2(1 + \mu )}, $$

where $G$, $\tau$, and $\gamma$ are the shear modulus, shear stress, and shear strain of the surrounding rock, respectively.

The shear modulus of the surrounding rock reinforcement body is given by the following:

22 $$ G_{R} = \frac{{\tau_{R} }}{\gamma } = \frac{{E_{R} }}{{2(1 + \mu_{R} )}}, $$

where $G_{R}$, $\tau_{R}$, and $\mu_{R}$ are the shear modulus, shear stress, and Poisson’s ratio of the surrounding rock reinforcement body, respectively.

According to Eqs. (21) and (22), Poisson’s ratio of the surrounding rock reinforcement body can be written as follows:

23 $$ \mu_{R} = (1 + \mu ) \times \frac{\tau }{{\tau_{R} }} \times \frac{{E_{R} }}{E} - 1. $$

According to Fig. 5, the shear stress ratio of the surrounding rock under load before and after the anchor reinforcement is introduced is as follows:

24 $$ \frac{\tau }{{\tau_{R} }} = \frac{C + q\tan \phi }{{C_{R} + q\tan \phi }}. $$

According to Eq. (17), the elastic modulus of the surrounding rock before and after the reinforcement can be derived by substituting Eq. (24) into Eq. (23). The following equation is obtained:

25 $$ \mu_{R} = \frac{C + q\tan \phi }{{C_{R} + q\tan \phi }} \times \frac{{{4}{\text{.64}}ql^{4} \pi (1 + \mu )}}{{4.64ql^{4} \pi - 3.39bh^{3} F_{3} (1 - \mu^{2} )}} - 1. $$

Based on the foregoing analysis, the strength parameters, $C_{R}$, $E_{R}$, and $\mu_{R}$, of the surrounding rock after bolt reinforcement can be obtained, and the mechanical parameters of the surrounding rock reinforcement body can be obtained by substituting the strength parameters into the theoretical formula presented in Section “Analysis of mechanical characteristics of surrounding rock of roadway roof without support”.

### Stability evaluation of surrounding rock reinforcement body

The theory of surrounding rock plus solid support considers the reinforcement effect of bolts. It further considers the bolt and surrounding rock as a whole to constrain deep surrounding rock. Bolt reinforcement improves the strength parameters and reflects the improvement in the mechanical parameters of the surrounding rock. Therefore, the stability of surrounding rock of a roadway is reflected by the relationship between the ultimate load that can be borne by the roof beam and load of overlying rock, as follows:

26 $$ K = 1 - \frac{q}{q*}, $$

where $K$ is the system stability coefficient of surrounding rock.

When $0 < K < {1}$, the overburden rock load is less than the ultimate load that the beam can bear; the closer the $K$ value is to 1, the better the stability of the surrounding rock. When $K < 0$, the overburden rock load is greater than the ultimate load that the beam can bear; hence, the surrounding rock is unstable.

The maximum axial stress of the surrounding rock reinforcement body anchor must not exceed the ultimate tensile strength of the bolt. Moreover, the maximum shear stress of the bolt must not exceed the anchoring strength of the surrounding rock. Thus, the bolt must satisfy the following conditions:

27 $$ \frac{{F_{3} S}}{A} \le \left[ \sigma \right], $$

where $\left[ \sigma \right]$ is the allowable axial stress of bolt materials.

28 $$ k\Delta w \le \left[ \tau \right], $$

where $k$ and $\left[ \tau \right]$ are the shear stiffness and allowable anchoring strength of the surrounding rock, respectively.

When Eqs. (27) and (28) are not satisfied, the stability coefficient of the surrounding rock reinforcement body is equal to that of the surrounding rock without reinforcement.

## Engineering case analysis

### Engineering background

**Figure 6:**
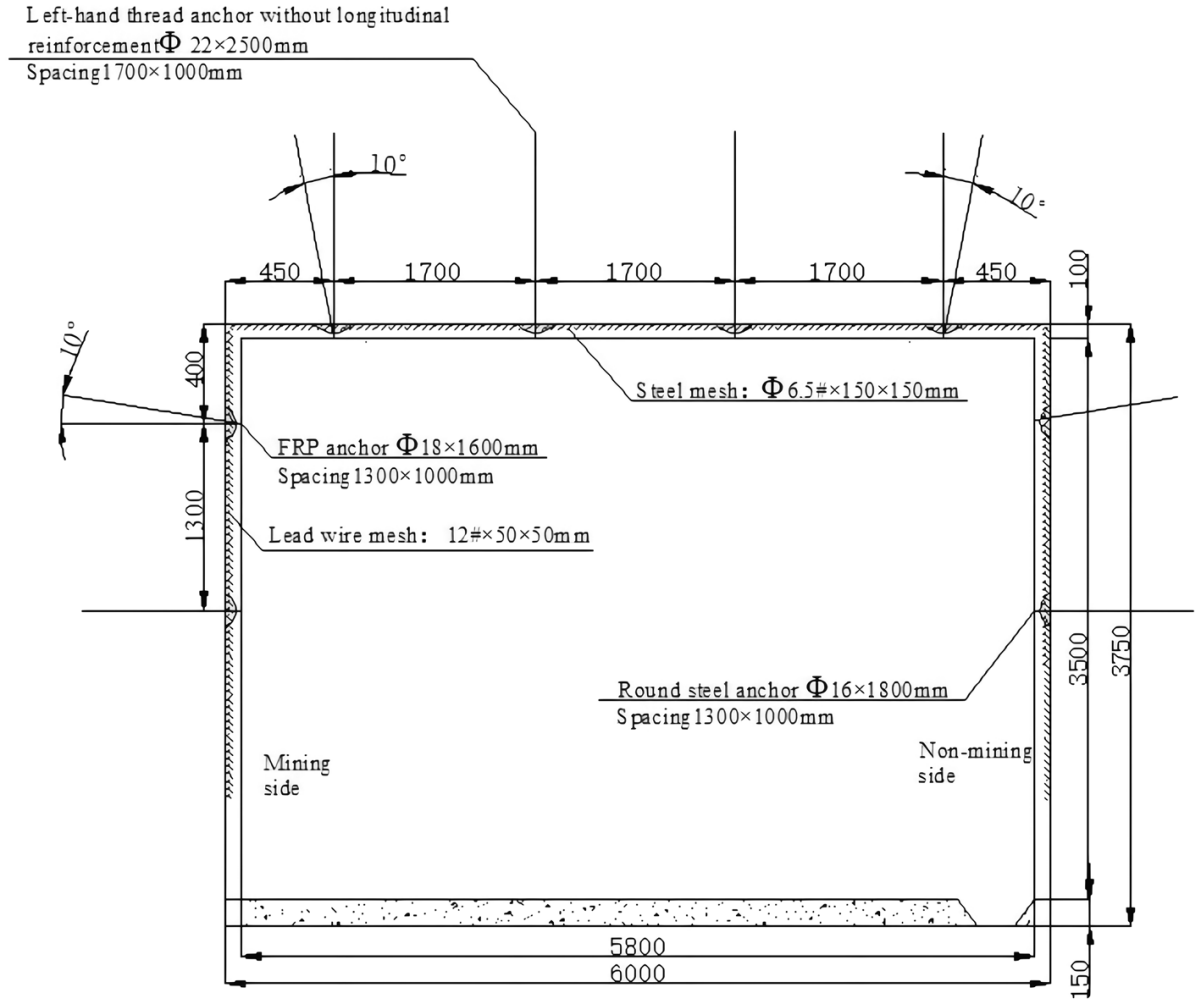
Roadway support drawing.

Ningtiaota coalmine is located in the middle of Shenmu County, Yulin City, Shaanxi Province, China. The average buried depth of the transportation roadway in coal mine S12001 is 200 m. The roadway section size is 6 m wide and 3.75 m high, and the average coal seam thickness is 4.3 m. The old roof, mainly coarse-grained sandstone, is generally 12.90 m thick. The direct roof, mainly fine-grained sandstone, is generally 9.55 m thick. The direct bottom, mainly carbonaceous mudstone, is generally 8.75 m thick. The old bottom is mainly fine sandstone. The physical and mechanical parameters of the rock strata are summarized in Table 1. Roadway support parameters are shown in Fig. 6.

**Table 1:** Physical and mechanical parameters of coal and rock mass.

Rock formation name	Volume weight/(KN·m^-3^)	Elastic Modulus/(GPa)	Poisson’s ratio	Friction angle/(°)	Cohesion/(MPa)
Sandy soil	18	3	0.38	15	0.5
Coarse sandstone	26	5.2	0.2	32	5.2
Fine sandstone	24	5.4	0.32	38	7.56
Coalseam	17	2	0.35	26	2.4
Carbonaceous mudstone	22	3.3	0.28	27	3.3

### Theoretical verification

The theoretical calculation formula and FLAC3D finite difference software are used to analyze the surrounding rock deformation of roadway roof. Then, the theoretical analytical solution is compared with the numerical simulation solution and actual monitoring data to verify the rationality of the derived analytical formula.

**Figure 7:**
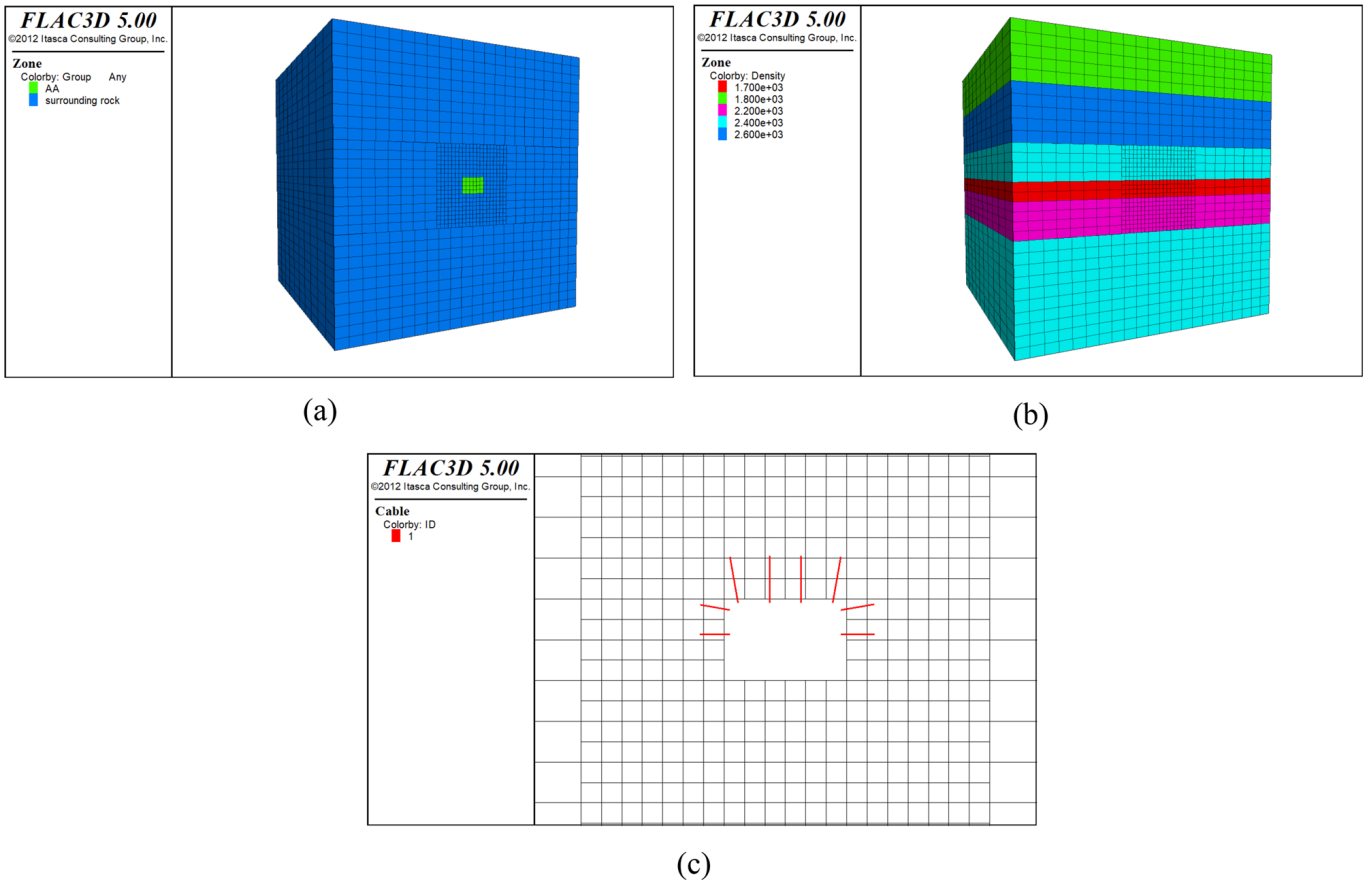
Numerical calculation model of roadway: (**a**) Meshing and grouping; (**b**) Density distribution; (**c**) Suspension roof support.

The size of the numerical simulation model is five times the diameter range of the chamber. The deformation boundary of the fundamental model is ${76}\,{{m}}\,{{ \times }}\,{76}\,{{m}}\,{{ \times }}\,{76}\,{{m}}$ considering the influence of excavation on the stress redistribution of the surrounding rock of the chamber. The boundary conditions of the model are as follows: the bottom and surroundings of the roadway are fixed boundaries; the top of the model is a free boundary. The roadway section is 3.75 ${\text{m}}$ high and 6.0 ${\text{m}}$ wide. Because the average buried depth of the excavation roadway is 200 ${\text{m}}$, the load acting on the model boundary is ${{q}}\,{ = }\,{2500}\,{{kg/m}}^{{3}} \,{{ \times }}\,{10}{{.0}}\,{{N/kg \times 164 m = 4}}{\text{.1 MPa}}$. The mesh of the surrounding rock near the roadway is closely spaced. The established roadway model is shown in Fig. 7.

**Figure 8:**
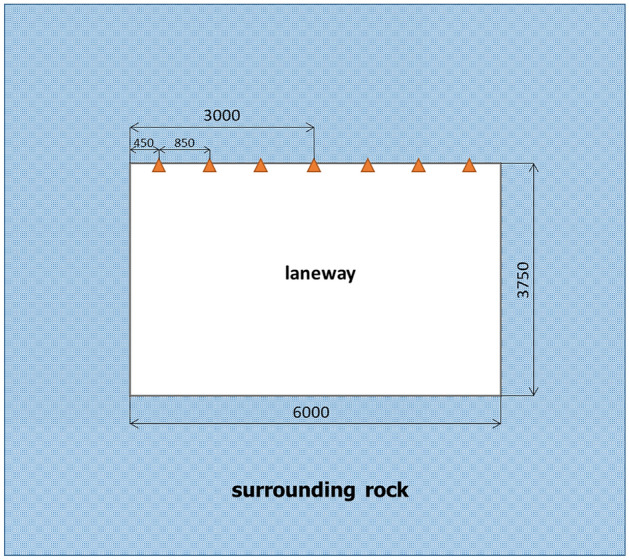
Cloud diagram of deformation of surrounding rock of roadway: (**a**) Cloud image of deformation of surrounding rock without support; (**b**) Cloud image of deformation of surrounding rock with support.

**Figure 9:**
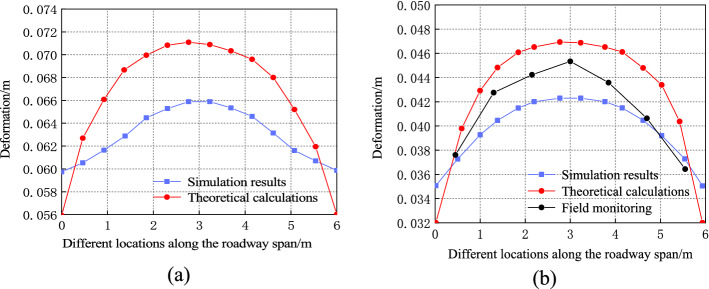
Comparisons of deformation curves of surrounding rock of roadway: (**a**) Comparison of deformation curves of roof surrounding rock without support; (**b**) Comparison of deformation curves of roof surrounding rock with roadway support.

The cloud map of the displacement of the simulated surrounding rock when the roadway is unsupported and supported is shown in Fig. 8. The theoretical calculation formula indicates that the deformation curve of the surrounding rock of the roadway roof can be obtained. The theoretical calculation and numerical simulation results are compared and analyzed, as shown in Fig. 9.

As presented in Fig. 9, the settlement values are not equal at different positions on the surface of the surrounding rock of the roof. The largest deformation occurs at the middle part of the surrounding rock of the roof and gradually decreases toward the two sides of the roof. The numerical simulation shows that the deformation law of the roadway roof is consistent with that reflected by the theoretical calculation. When the surrounding rock is unsupported, the deformation values of the middle part of the surrounding rock of the roof obtained using the theoretical formula and numerical simulation are 0.071 and 0.066 ${\text{m}}$, respectively. When the surrounding rock is supported, the deformation values are 0.047 and 0.042 ${\text{m}}$, respectively. Because of the support, the roof deformation values given by the theoretical calculation and FLAC3D numerical simulation compared with the deformation before the support was introduced were reduced. This indicates that the anchor rod strengthens the roof, improves the self-stabilizing ability of the surrounding rock, and stabilizes the roadway. Moreover, the calculated values approximate the results of the FLAC3D simulation, demonstrating the rationality of the established model.

**Figure 10:**
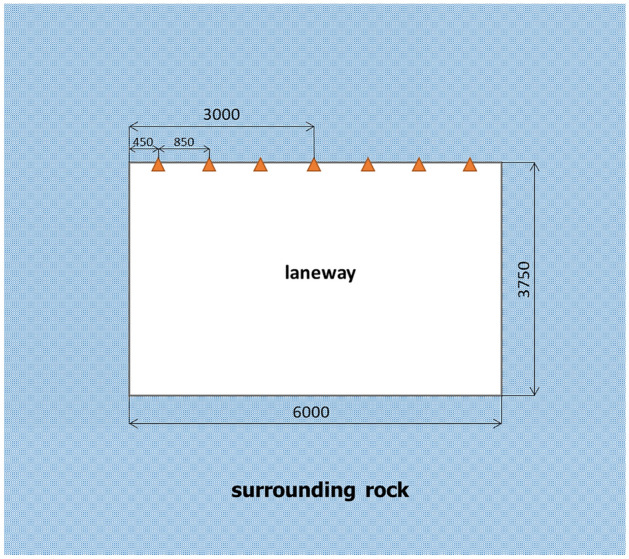
Schematic of roadway section monitoring site locations.

**Figure 11:**
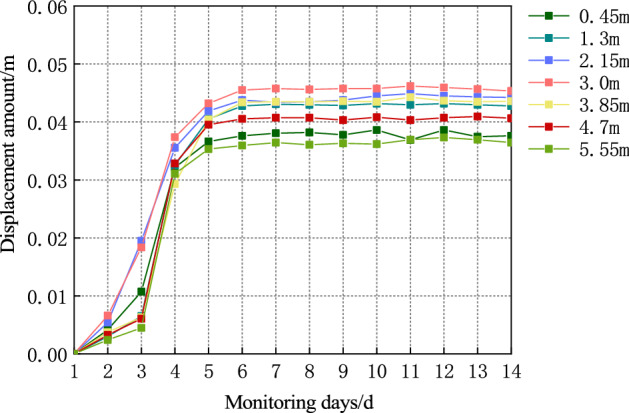
Monitoring diagram of surrounding rock displacement of roadway roof.

The site monitoring stations are arranged along the roadway of the S12001 belt transportation working face of Ningtiaota coalmine; the installation and burial of observation points follow the working face. Monitoring the displacement of surrounding rock on the roadway surface is accomplished with the JSS30A convergence meter. Convergent meters such as the JSS30A show a digital display of the displacements between two points, and are usually used to monitor the change in distance between the two points. The measuring points of the convergence meter are installed 0.45, 1.3, 2.15, 3, 3.85, 4.7 and 5.55 ${\text{m}}$ away from the left side, as shown in Fig. 10. The surface displacement curve of the surrounding rock of the roadway roof monitored on-site is shown in Fig. 11.

The field monitoring results are compared with the theoretical calculation results, as shown in Fig. 9b. The figure indicates that the maximum surface deformation of the surrounding rock of roof obtained by the on-site monitoring of the roadway surrounding rock with support is 0.045${\text{m}}$, approximating the theoretical value of 0.047 ${\text{m}}$; this further verifies the applicability of the theory.

#### Stability evaluation of surrounding rock

By substituting the roadway surrounding rock parameters and bolt support parameters into Eqs. (14)–(25), the mechanical parameters of the surrounding rock reinforcement body can be obtained; the values are summarized in Table 2.

**Table 2:** Changes in physical and mechanical parameters of surrounding rock of roadway roof.

Mechanical parameters of surrounding rock		Mechanical parameters of surrounding rock reinforcement body	
Parameter	Value	Parameter	Value
$E{\text{/GPa}}$	5.4	$E_{R} {\text{/GPa}}$	5.401
$C{\text{/MPa}}$	7.56	$C_{R} {\text{/MPa}}$	7.61
$\mu$	0.32	$\mu_{R}$	0.314

The mechanical parameters of the surrounding rock of the roadway roof with and without support are compared in Table 2. For the surrounding rock with support, the elastic modulus and cohesion increase by 0.02 and 0.67%, respectively, and Poisson’s ratio decreases by 1.88%. The stability coefficient of the surrounding rock, $K = {0}.74 > {0}$, indicates good surrounding rock stability. Moreover, the maximum axial force and maximum shear force on the bolt are $\sigma {\text{ = 164 N/mm}}^{{2}} \le {\text{ 300 N/mm}}{}^{{2}} \,$ and $\tau { = 2}{\text{.51 N/mm}}^{{2}} \, \le {\text{3 N/mm}}^{{2}}$, respectively; thus, the bolt support design is reasonable.

## Analysis of anchor support parameters on mechanical parameters and stability of surrounding rock reinforcement body

### Analysis of influence of bolt support parameters on mechanical parameters of surrounding rock reinforcement body

**Figure 12:**
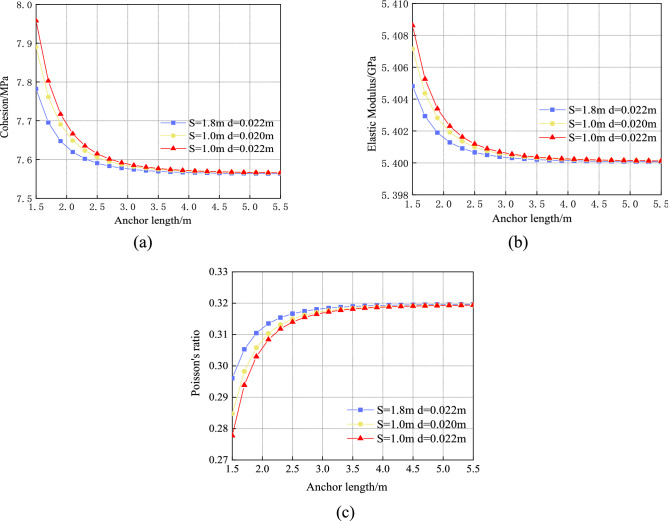
Influence of anchor length, spacing, and diameter on physical and mechanical parameters of surrounding rock reinforcement body: (**a**)Influence on cohesion; (**b**) Influence on elastic modulus; (**c**) Influence on Poisson's ratio.

The changes in the mechanical characteristics of the surrounding rock reinforcement body considering different lengths, spacings, and diameters of the anchor rod as well as the engineering geological parameters of the surrounding rock of S12001 belt transportation roadway are shown in Fig. 12. The figure shows that the physical and mechanical parameters of the surrounding rock have improved to a certain extent. When the anchor spacing and diameter are 1 and 0.022 ${\text{m}}$, respectively, and the length increases from 1.5 to 5.5 ${\text{m}}$, the cohesion, elastic modulus, and Poisson’s ratio of the surrounding rock decrease by 5.0, 0.16 and 14.75%, respectively. The physical and mechanical parameters of the surrounding rock reinforcement body tend to remain in the original rock state mainly because the scope of the reinforced surrounding rock increases with the anchor length. After an equivalent conversion, the nature of the surrounding rock reinforcement body is similar to the original rock state.

When the bolt diameter and length are 0.022 and 1.5 ${\text{m}}$, respectively, the surrounding rock cohesion decreases by 2.21%, the elastic modulus decreases by 0.07%, and Poisson’s ratio increases by 6.51% as the anchor rod spacing increases from 1.0 to 1.8 m. When the anchor rod spacing is 1.0 ${\text{m}}$ and the bolt length is 1.5 ${\text{m}}$, the cohesion of the surrounding rock increases by 0.86%, the elastic modulus increases by 0.03%, and Poisson's ratio decreases by 2.37% as the anchor rod diameter increases from 0.020 to 0.022 ${\text{m}}$. Anchor length and spacing have a greater effect on the physical and mechanical parameters of the surrounding rock reinforcement body than the anchor diameter.

**Figure 13:**
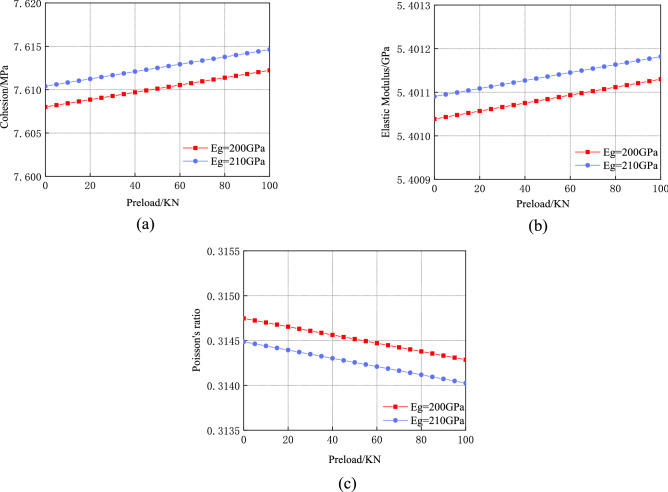
Influence of pre-tightening force and elastic modulus of anchor on physical and mechanical parameters of surrounding rock reinforcement body: (**a**) Influence on cohesion; (**b**) Influence on elastic modulus; (**c**) Influence on Poisson’s ratio.

The changes in the physical and mechanical parameters of the surrounding rock reinforcement body under the different pretension forces and anchor rod elastic modulus conditions are shown in Fig. 13.

As shown in the figure, when the elastic modulus of the anchor is 210 ${\text{GPa}}$ and the preload increases from 0 to 100 ${\text{kN}}$, the cohesion and elastic modulus increase by 0.06% and 0.01%, respectively, and Poisson’s ratio decreases by 0.15%. Furthermore, when the elastic modulus of the anchor increases from 200 to 210 ${\text{GPa}}$ and the preload is 100 ${\text{kN}}$, the cohesion and elastic modulus increase by 0.03% and 0.01%, respectively, and Poisson's ratio decreases by 0.89%.

### Analysis of influence of anchor parameters on surrounding rock reinforcement body

**Figure 14:**
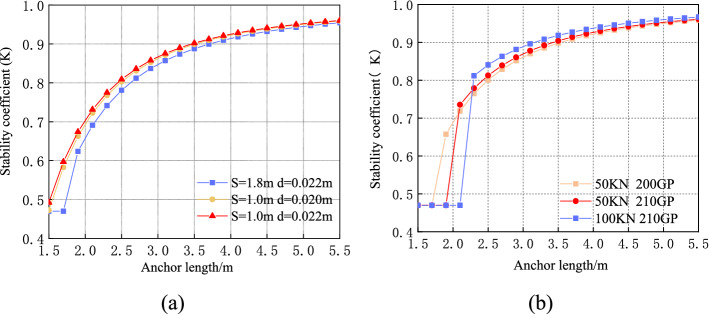
Effect of different values of anchor rod length, spacing, diameter, elastic modulus, and pretension force on stability of surrounding rock reinforcement body: (**a**) Different values of anchor length, diameter, and spacing; (**b**) Different values of anchor length, pretension force, and elastic modulus.

The changes in the stability of the surrounding rock considering different lengths, spacings, and diameters of the anchor bolt are shown in Fig. 14a. In this case, the engineering geological parameters of the surrounding rock of the S12001 transport channel roadway and other conditions are unchanged. The figure indicates that as the anchor length increases, the surrounding rock stability coefficient, $K$, increases until it gradually tends to 0.96. Although the physical and mechanical parameters of the surrounding rock reinforcement body under the long anchor support are smaller than those of the short anchor support, the stability increases due to the large reinforcement range of the surrounding rock.

When the anchor spacing and diameter are 1.8 and 0.022, respectively, and the anchor length is less than 1.9 ${\text{m}}$, the stress on the anchor exceeds the allowable stress, and the surrounding rock fails. The stability coefficient of the supported surrounding rock must be equal to the stability coefficient when it is unsupported (i.e., $K$ is 0.47). When the anchor rod spacing and diameter are 1 and 0.022 ${\text{m}}$, respectively, and as the anchor rod length increases from 1.5 to 5.5 ${\text{m}}$, $K$ increases by 0.46. When the diameter and length of the anchor rod are 0.022 and 2.1 ${\text{m}}$, respectively, and the anchor rod spacing decreases from 1.8 to 1.0 ${\text{m}}$, $K$ increases by 0.04. When the anchor rod spacing and length are 1.0 and 2.1 ${\text{m}}$, respectively, and the anchor rod diameter increases from 0.020 to 0.022 ${\text{m}}$, $K$ increases by 0.01. Therefore, when the design anchor length is short, the anchor spacing can be reduced or the anchor diameter can be increased to improve the stability of the surrounding rock. Under the premise that stability is ensured, when the anchor rod length is long, the anchor rod spacing can be increased to reduce the amount of anchor rod material and thus avoid waste.

The change in the stability of the surrounding rock when the anchor length, pretension force, and elastic modulus as well as other conditions are unchanged is shown in Fig. 14b. The figure indicates that the increase in the pretension force improves the stability of the surrounding rock. From the perspective of strengthening the surrounding rock, the general presumption is that the greater the pretension force of the anchor rod, the better the stability of the surrounding rock; however, the application of the anchor rod pretension force has a certain limit. When the bolt elastic modulus and pretension force are 210 ${\text{GPa}}$ and 50 ${\text{kN}}$, respectively, and the bolt length is less than 1.9 ${\text{m}}$, the bolt stress exceeds the allowable stress. Moreover, the bolt fails when the stability coefficient of the surrounding rock is equal to that when support is not applied; $K$ is 0.47. Similarly, the bolt fails when the anchor pretension force increases to 100 ${\text{kN}}$ and the anchor length is less than 2.1 ${\text{m}}$ with $K$ equal to 0.47. When the anchor pretension force and anchor length are 50 ${\text{kN}}$ and 2.1 ${\text{m}}$, respectively, the $K$ value increases by 0.02 with the elastic factor ranging from 200 to 210 ${\text{GPa}}$. Therefore, in bolt design, the shorter the bolt length, the smaller the bolt pretension force. When the anchor rod length is short and the pre-tightening force is large, an anchor rod with a large elastic modulus can be selected to improve the stability of the surrounding rock.

## Conclusion

1. A roof beam model was established according to the deformation law of surrounding rock of rectangular roadway roof. Moreover, elastic–plastic analysis was implemented to derive the deflection formula of the roof beam model in the plastic state without support and the ultimate load that the beam can bear.
2. The model of surrounding rock reinforcement body is developed considering the influence of changes in the anchor rod on the mechanical state of the surrounding rock. The expressions of the elastic modulus, Poisson's ratio, and cohesive force of surrounding rock reinforcement body are derived. Based on the foregoing, the mechanical characteristics of supported surrounding rock are obtained. Accordingly, a method for evaluating the roadway surrounding rock stability with the limit load of the surrounding rock reinforcement body is proposed, and the surrounding rock stability coefficient is defined.
3. The deformation curves of the surrounding rock of the roadway roof with and without support are analyzed. The theory is verified by FLAC3D, field monitoring, and actual engineering cases. The results show that the theoretical calculations are consistent with the numerical simulation and field monitoring results, verifying the rationality of the theory.
4. The influence of the anchor rod support parameters on the physical and mechanical properties of the surrounding rock reinforcement body is analyzed considering the actual engineering cases. The results show that the physical and mechanical properties of the surrounding rock reinforcement body are more affected by the changes in bolt length and spacing than by the changes in the preload, elastic modulus, and diameter of the bolt. Hence, bolt length and spacing must be reasonably designed in practical engineering.
5. The present study also demonstrates that to improve the stability of the surrounding rock, the design of the anchor length and spacing must apply the following principle: the bolt must either be long and sparsely spaced or short and densely spaced. As indicated by the stability coefficient, the increase in anchor length tends to improve surrounding rock stability. At this point, optimizing the anchor length and spacing must be considered to reduce the amount of anchor material and thus avoid material waste. Moreover, the shorter the anchor length, the smaller the anchor pre-tightening force must be. When the anchor length is short but the pre-tightening force is considerable, a bolt with a high elastic modulus can be selected to improve the stability of the surrounding rock.

## Supplementary Information

Supplementary Information.

## Data Availability

The datasets used or analysed during the current study available from the corresponding author on reasonable request.
