# Supplementary material for: Mechanical characteristics and stability analysis of surrounding rock reinforcement in rectangular roadway
Source: Sci Rep. 2022 Dec 23;12:22234. doi: 10.1038/s41598-022-26773-z (PMC9789071; doi:10.1038/s41598-022-26773-z)
Supplement: Supplementary file 1 — Supplementary Information. [file 41598_2022_26773_MOESM1_ESM.doc]

Data availability

The datasets used and/or analysed during the current study available from the corresponding author on reasonable request.

| Raw data for Figure 9(a) | | |
| --- | --- | --- |
| X | Y(Simulation results) | Y(Theoretical calculations) |
| 0.00 | 0.0598 | 0.0559 |
| 0.50 | 0.0605 | 0.0627 |
| 1.00 | 0.0617 | 0.0661 |
| 1.38 | 0.0629 | 0.0687 |
| 1.85 | 0.0645 | 0.0700 |
| 2.20 | 0.0653 | 0.0708 |
| 2.77 | 0.0659 | 0.0711 |
| 3.23 | 0.0659 | 0.0709 |
| 3.77 | 0.0653 | 0.0703 |
| 4.15 | 0.0646 | 0.0696 |
| 4.62 | 0.0632 | 0.0680 |
| 5.03 | 0.0616 | 0.0652 |
| 5.54 | 0.0607 | 0.0620 |
| 6.00 | 0.0599 | 0.0560 |

| Raw data for Figure 9(b) | | | | |
| --- | --- | --- | --- | --- |
| X | Y(Simulation results) | Y(Theoretical calculations) | X | Y(Field monitoring) |
| 0.00 | 0.0351 | 0.0320 | 0.45 | 0.038 |
| 0.50 | 0.0373 | 0.0398 | 1.30 | 0.043 |
| 1.00 | 0.0393 | 0.0429 | 2.15 | 0.044 |
| 1.38 | 0.0405 | 0.0448 | 3.00 | 0.045 |
| 1.85 | 0.0415 | 0.0461 | 3.85 | 0.044 |
| 2.20 | 0.0420 | 0.0465 | 4.70 | 0.041 |
| 2.77 | 0.0423 | 0.0469 | 5.55 | 0.036 |
| 3.23 | 0.0423 | 0.0469 |  |  |
| 3.77 | 0.0420 | 0.0465 |  |  |
| 4.15 | 0.0415 | 0.0461 |  |  |
| 4.62 | 0.0405 | 0.0448 |  |  |
| 5.03 | 0.0392 | 0.0434 |  |  |
| 5.54 | 0.0373 | 0.0404 |  |  |
| 6.00 | 0.0351 | 0.0320 |  |  |

| Raw data for Figure 11 | | | | | | | |
| --- | --- | --- | --- | --- | --- | --- | --- |
| Observation  Points  Monitoring  Days | 0.45 | 1.3 | 2.15 | 3.0 | 3.85 | 4.7 | 5.55 |
| 1.00 | 0.000 | 0.000 | 0.000 | 0.000 | 0.000 | 0.000 | 0.000 |
| 2.00 | 0.004 | 0.003 | 0.005 | 0.007 | 0.004 | 0.003 | 0.002 |
| 3.00 | 0.011 | 0.007 | 0.020 | 0.018 | 0.006 | 0.006 | 0.004 |
| 4.00 | 0.032 | 0.033 | 0.036 | 0.037 | 0.029 | 0.033 | 0.031 |
| 5.00 | 0.037 | 0.041 | 0.042 | 0.043 | 0.040 | 0.040 | 0.035 |
| 6.00 | 0.038 | 0.043 | 0.044 | 0.046 | 0.043 | 0.041 | 0.036 |
| 7.00 | 0.038 | 0.043 | 0.043 | 0.046 | 0.043 | 0.041 | 0.036 |
| 8.00 | 0.038 | 0.043 | 0.043 | 0.046 | 0.043 | 0.041 | 0.036 |
| 9.00 | 0.038 | 0.043 | 0.044 | 0.046 | 0.044 | 0.040 | 0.036 |
| 10.00 | 0.039 | 0.043 | 0.045 | 0.046 | 0.043 | 0.041 | 0.036 |
| 11.00 | 0.037 | 0.043 | 0.045 | 0.046 | 0.044 | 0.040 | 0.037 |
| 12.00 | 0.039 | 0.043 | 0.045 | 0.046 | 0.044 | 0.041 | 0.037 |
| 13.00 | 0.037 | 0.043 | 0.044 | 0.046 | 0.043 | 0.041 | 0.037 |
| 14.00 | 0.038 | 0.043 | 0.044 | 0.045 | 0.044 | 0.041 | 0.036 |

| Raw data for Figure 12(a) | | | |
| --- | --- | --- | --- |
| Anchor length | S=1.0m d=0.022m | S=1.0m d=0.020m | S=1.8m d=0.022m |
| 1.50 | 7.958 | 7.890 | 7.782 |
| 1.70 | 7.803 | 7.762 | 7.695 |
| 1.90 | 7.717 | 7.691 | 7.648 |
| 2.10 | 7.666 | 7.649 | 7.619 |
| 2.30 | 7.635 | 7.623 | 7.602 |
| 2.50 | 7.615 | 7.606 | 7.591 |
| 2.70 | 7.601 | 7.595 | 7.583 |
| 2.90 | 7.592 | 7.587 | 7.578 |
| 3.10 | 7.585 | 7.582 | 7.574 |
| 3.30 | 7.581 | 7.578 | 7.571 |
| 3.50 | 7.577 | 7.575 | 7.569 |
| 3.70 | 7.574 | 7.572 | 7.568 |
| 3.90 | 7.572 | 7.571 | 7.567 |
| 4.10 | 7.571 | 7.569 | 7.566 |
| 4.30 | 7.569 | 7.568 | 7.565 |
| 4.50 | 7.568 | 7.568 | 7.565 |
| 4.70 | 7.568 | 7.567 | 7.564 |
| 4.90 | 7.567 | 7.566 | 7.564 |
| 5.10 | 7.567 | 7.566 | 7.564 |
| 5.30 | 7.566 | 7.566 | 7.563 |
| 5.50 | 7.566 | 7.565 | 7.563 |

| Raw data for Figure 12(b) | | | |
| --- | --- | --- | --- |
| Anchor length | S=1.0m d=0.022m | S=1.0m d=0.020m | S=1.8m d=0.022m |
| 1.50 | 5.409 | 5.407 | 5.405 |
| 1.70 | 5.405 | 5.404 | 5.403 |
| 1.90 | 5.403 | 5.403 | 5.402 |
| 2.10 | 5.402 | 5.402 | 5.401 |
| 2.30 | 5.402 | 5.401 | 5.401 |
| 2.50 | 5.401 | 5.401 | 5.401 |
| 2.70 | 5.401 | 5.401 | 5.401 |
| 2.90 | 5.401 | 5.401 | 5.400 |
| 3.10 | 5.401 | 5.400 | 5.400 |
| 3.30 | 5.400 | 5.400 | 5.400 |
| 3.50 | 5.400 | 5.400 | 5.400 |
| 3.70 | 5.400 | 5.400 | 5.400 |
| 3.90 | 5.400 | 5.400 | 5.400 |
| 4.10 | 5.400 | 5.400 | 5.400 |
| 4.30 | 5.400 | 5.400 | 5.400 |
| 4.50 | 5.400 | 5.400 | 5.400 |
| 4.70 | 5.400 | 5.400 | 5.400 |
| 4.90 | 5.400 | 5.400 | 5.400 |
| 5.10 | 5.400 | 5.400 | 5.400 |
| 5.30 | 5.400 | 5.400 | 5.400 |
| 5.50 | 5.400 | 5.400 | 5.400 |

| Raw data for Figure 12(c) | | | |
| --- | --- | --- | --- |
| Anchor length | S=1.0m d=0.022m | S=1.0m d=0.020m | S=1.8m d=0.022m |
| 1.50 | 0.278 | 0.285 | 0.296 |
| 1.70 | 0.294 | 0.298 | 0.305 |
| 1.90 | 0.303 | 0.306 | 0.310 |
| 2.10 | 0.308 | 0.310 | 0.314 |
| 2.30 | 0.312 | 0.313 | 0.315 |
| 2.50 | 0.314 | 0.315 | 0.317 |
| 2.70 | 0.315 | 0.316 | 0.317 |
| 2.90 | 0.317 | 0.317 | 0.318 |
| 3.10 | 0.317 | 0.318 | 0.318 |
| 3.30 | 0.318 | 0.318 | 0.319 |
| 3.50 | 0.318 | 0.318 | 0.319 |
| 3.70 | 0.318 | 0.319 | 0.319 |
| 3.90 | 0.319 | 0.319 | 0.319 |
| 4.10 | 0.319 | 0.319 | 0.319 |
| 4.30 | 0.319 | 0.319 | 0.319 |
| 4.50 | 0.319 | 0.319 | 0.319 |
| 4.70 | 0.319 | 0.319 | 0.320 |
| 4.90 | 0.319 | 0.319 | 0.320 |
| 5.10 | 0.319 | 0.319 | 0.320 |
| 5.30 | 0.319 | 0.319 | 0.320 |
| 5.50 | 0.319 | 0.319 | 0.320 |

| Raw data for Figure 13(a) | | |
| --- | --- | --- |
| Preload | Eg=200GPa | Eg=210GPa |
| 0.00 | 7.60801 | 7.61040 |
| 5.00 | 7.60822 | 7.61061 |
| 10.00 | 7.60843 | 7.61082 |
| 15.00 | 7.60864 | 7.61103 |
| 20.00 | 7.60885 | 7.61124 |
| 25.00 | 7.60906 | 7.61145 |
| 30.00 | 7.60927 | 7.61166 |
| 35.00 | 7.60948 | 7.61187 |
| 40.00 | 7.60970 | 7.61209 |
| 45.00 | 7.60991 | 7.61230 |
| 50.00 | 7.61012 | 7.61251 |
| 55.00 | 7.61033 | 7.61272 |
| 60.00 | 7.61054 | 7.61293 |
| 65.00 | 7.61075 | 7.61314 |
| 70.00 | 7.61096 | 7.61335 |
| 75.00 | 7.61117 | 7.61356 |
| 80.00 | 7.61139 | 7.61378 |
| 85.00 | 7.61160 | 7.61399 |
| 90.00 | 7.61181 | 7.61420 |
| 95.00 | 7.61202 | 7.61441 |
| 100.00 | 7.61223 | 7.61462 |

| Raw data for Figure 13(b) | | |
| --- | --- | --- |
| Preload | Eg=200GPa | Eg=210GPa |
| 0.00 | 5.40104 | 5.40109 |
| 5.00 | 5.40104 | 5.40109 |
| 10.00 | 5.40105 | 5.40110 |
| 15.00 | 5.40105 | 5.40110 |
| 20.00 | 5.40106 | 5.40111 |
| 25.00 | 5.40106 | 5.40111 |
| 30.00 | 5.40107 | 5.40112 |
| 35.00 | 5.40107 | 5.40112 |
| 40.00 | 5.40108 | 5.40113 |
| 45.00 | 5.40108 | 5.40113 |
| 50.00 | 5.40108 | 5.40114 |
| 55.00 | 5.40109 | 5.40114 |
| 60.00 | 5.40109 | 5.40115 |
| 65.00 | 5.40110 | 5.40115 |
| 70.00 | 5.40110 | 5.40115 |
| 75.00 | 5.40111 | 5.40116 |
| 80.00 | 5.40111 | 5.40116 |
| 85.00 | 5.40112 | 5.40117 |
| 90.00 | 5.40112 | 5.40117 |
| 95.00 | 5.40113 | 5.40118 |
| 100.00 | 5.40113 | 5.40118 |

| Raw data for Figure 13(c) | | |
| --- | --- | --- |
| Preload | Eg=200GPa | Eg=210GPa |
| 0.00 | 0.31475 | 0.31449 |
| 5.00 | 0.31472 | 0.31446 |
| 10.00 | 0.31470 | 0.31444 |
| 15.00 | 0.31468 | 0.31442 |
| 20.00 | 0.31465 | 0.31439 |
| 25.00 | 0.31463 | 0.31437 |
| 30.00 | 0.31461 | 0.31435 |
| 35.00 | 0.31459 | 0.31432 |
| 40.00 | 0.31456 | 0.31430 |
| 45.00 | 0.31454 | 0.31428 |
| 50.00 | 0.31452 | 0.31426 |
| 55.00 | 0.31449 | 0.31423 |
| 60.00 | 0.31447 | 0.31421 |
| 65.00 | 0.31445 | 0.31419 |
| 70.00 | 0.31442 | 0.31416 |
| 75.00 | 0.31440 | 0.31414 |
| 80.00 | 0.31438 | 0.31412 |
| 85.00 | 0.31436 | 0.31409 |
| 90.00 | 0.31433 | 0.31407 |
| 95.00 | 0.31431 | 0.31405 |
| 100.00 | 0.31429 | 0.31403 |

| Raw data for Figure 14(a) | | | |
| --- | --- | --- | --- |
| Anchor length | S=1.0m d=0.022m | S=1.0m d=0.020m | S=1.8m d=0.022m |
| 1.50 | 0.492 | 0.473 | 0.470 |
| 1.70 | 0.597 | 0.583 | 0.470 |
| 1.90 | 0.674 | 0.663 | 0.624 |
| 2.10 | 0.731 | 0.723 | 0.691 |
| 2.30 | 0.775 | 0.768 | 0.742 |
| 2.50 | 0.809 | 0.803 | 0.781 |
| 2.70 | 0.836 | 0.831 | 0.812 |
| 2.90 | 0.858 | 0.853 | 0.837 |
| 3.10 | 0.875 | 0.872 | 0.857 |
| 3.30 | 0.890 | 0.887 | 0.874 |
| 3.50 | 0.902 | 0.899 | 0.888 |
| 3.70 | 0.912 | 0.910 | 0.900 |
| 3.90 | 0.921 | 0.919 | 0.910 |
| 4.10 | 0.929 | 0.926 | 0.918 |
| 4.30 | 0.935 | 0.933 | 0.926 |
| 4.50 | 0.941 | 0.939 | 0.932 |
| 4.70 | 0.946 | 0.944 | 0.938 |
| 4.90 | 0.950 | 0.948 | 0.943 |
| 5.10 | 0.954 | 0.952 | 0.947 |
| 5.30 | 0.957 | 0.956 | 0.951 |
| 5.50 | 0.960 | 0.959 | 0.955 |

| Raw data for Figure 14(b) | | | |
| --- | --- | --- | --- |
| Anchor length | 50KN 200GP | 50KN 210GP | 100KN 210GP |
| 1.50 | 0.47000 | 0.47000 | 0.4700 |
| 1.70 | 0.47000 | 0.47000 | 0.4700 |
| 1.90 | 0.65760 | 0.47000 | 0.4700 |
| 2.10 | 0.71867 | 0.73552 | 0.4700 |
| 2.30 | 0.76493 | 0.77902 | 0.8118 |
| 2.50 | 0.80073 | 0.81268 | 0.8405 |
| 2.70 | 0.82899 | 0.83925 | 0.8632 |
| 2.90 | 0.85166 | 0.86056 | 0.8813 |
| 3.10 | 0.87012 | 0.87791 | 0.8961 |
| 3.30 | 0.88535 | 0.89222 | 0.9083 |
| 3.50 | 0.89805 | 0.90416 | 0.9185 |
| 3.70 | 0.90875 | 0.91423 | 0.9270 |
| 3.90 | 0.91786 | 0.92279 | 0.9343 |
| 4.10 | 0.92567 | 0.93013 | 0.9406 |
| 4.30 | 0.93242 | 0.93647 | 0.9460 |
| 4.50 | 0.93829 | 0.94199 | 0.9506 |
| 4.70 | 0.94342 | 0.94682 | 0.9548 |
| 4.90 | 0.94795 | 0.95107 | 0.9584 |
| 5.10 | 0.95195 | 0.95483 | 0.9616 |
| 5.30 | 0.95550 | 0.95817 | 0.9644 |
| 5.50 | 0.95868 | 0.96116 | 0.9670 |
